# Calibrating the Bacterial Growth Rate Speedometer: A Re-evaluation of the Relationship Between Basal ppGpp, Growth, and RNA Synthesis in *Escherichia coli*

**DOI:** 10.3389/fmicb.2020.574872

**Published:** 2020-09-17

**Authors:** Nicole C. E. Imholz, Marek J. Noga, Niels J. F. van den Broek, Gregory Bokinsky

**Affiliations:** Department of Bionanoscience, Kavli Institute of Nanoscience, Delft University of Technology, Delft, Netherlands

**Keywords:** ppGpp, growth rate, RNA polymerase, RNA synthesis, *Escherichia coli*

## Abstract

The molecule guanosine tetraphophosphate (ppGpp) is most commonly considered an alarmone produced during acute stress. However, ppGpp is also present at low concentrations during steady-state growth. Whether ppGpp controls the same cellular targets at both low and high concentrations remains an open question and is vital for understanding growth rate regulation. It is widely assumed that basal ppGpp concentrations vary inversely with growth rate, and that the main function of basal ppGpp is to regulate transcription of ribosomal RNA in response to environmental conditions. Unfortunately, studies to confirm this relationship and to define regulatory targets of basal ppGpp are limited by difficulties in quantifying basal ppGpp. In this Perspective we compare reported concentrations of basal ppGpp in *E. coli* and quantify ppGpp within several strains using a recently developed analytical method. We find that although the inverse correlation between ppGpp and growth rate is robust across strains and analytical methods, absolute ppGpp concentrations do not absolutely determine RNA synthesis rates. In addition, we investigated the consequences of two separate RNA polymerase mutations that each individually reduce (but do not abolish) sensitivity to ppGpp and find that the relationship between ppGpp, growth rate, and RNA content of single-site mutants remains unaffected. Both literature and our new data suggest that environmental conditions may be communicated to RNA polymerase via an additional regulator. We conclude that basal ppGpp is one of potentially several agents controlling ribosome abundance and DNA replication initiation, but that evidence for additional roles in controlling macromolecular synthesis requires further study.

## Introduction

How might a bacteria cell measure its own growth rate? In the model bacterium *Escherichia coli*, the small molecule guanosine tetraphosphate (ppGpp) is closely tied to growth rate control. However, due to the circumstances of its discovery, ppGpp is more familiar as a stress or starvation signal. ppGpp and guanosine pentaphosphate (pppGpp), collectively called (p)ppGpp, were first identified in *E. coli* as compounds produced in strains that inhibit stable RNA synthesis upon amino acid starvation, a phenomenon known as the stringent response (Cashel and Gallant, 1969). The source of (p)ppGpp during the stringent response is the enzyme RelA, which synthesizes (p)ppGpp in response to uncharged (non-aminoacyl) tRNA binding the acceptor site of an actively translating ribosome (Haseltine and Block, 1973). The high concentrations of (p)ppGpp observed during acute starvation (600–1000 pmol OD^–1^ for ppGpp) (Harshman and Yamazaki, 1971; Lazzarini et al., 1971) drive profound responses via both transcriptional and post-translational mechanisms (Traxler et al., 2008; Kanjee et al., 2012). The overall result of the stringent response is strong inhibition of all macromolecule synthesis (rRNA, DNA, proteins, phospholipids, and peptidoglycan), leading to growth arrest. (p)ppGpp is hydrolyzed by the essential enzyme SpoT. SpoT also carries an active (p)ppGpp synthase domain, although the specific biochemical trigger of (p)ppGpp synthesis by SpoT has not yet been identified. As a recent study suggests that ppGpp is more potent than pppGpp in mediating growth rate control in *E. coli* (Mechold et al., 2013) we focus exclusively upon ppGpp.

Comparatively less well-understood are the functions of “basal ppGpp”: the ppGpp concentrations observed during steady-state growth in *E. coli* in the absence of stress (between 10 and 90 pmol OD^–1^). When growth rate is varied by nutritional quality, basal ppGpp correlates inversely with growth rate. Basal ppGpp is essential in minimal media as it is required to activate transcription of amino acid pathways (Traxler et al., 2011). Just as ppGpp strongly inhibits stable RNA synthesis at high concentrations, basal ppGpp mildly inhibits transcriptional initiation from ribosomal RNA (rRNA) promoters, thus at least partly determining ribosome abundance during steady-state growth (Ryals et al., 1982). The inverse relationship between ppGpp and growth rate is thought to reflect the rheostat-like function of ppGpp as a regulator of ribosomal biosynthesis in response to nutrient availability. If ppGpp is artificially deviated from natural basal concentrations, growth is slowed, suggesting that ppGpp finds a growth-optimum level and adjusts rRNA expression accordingly (Zhu and Dai, 2019). The rate of DNA replication initiation also adjusts to small increases in basal ppGpp (Schreiber et al., 1995), and a strain entirely lacking ppGpp [*relA^–^ spoT^–^*, or (p)ppGpp^0^] does not vary DNA replication initiation in response to growth rate, suggesting that ppGpp participates in regulating the DNA-biomass ratio (Fernández-Coll et al., 2020). As ribosome abundance is one of the factors determining the global protein synthesis rate, ppGpp may act as a growth rate-reporting signal that smoothly adjusts the steady-state rate of overall biomass synthesis (Hui et al., 2015). ppGpp should be thus considered a growth rate speedometer as well as a stress signal.

The observation that high ppGpp concentrations inhibit biomass synthesis suggests that basal ppGpp concentrations might also directly regulate all biomass synthesis pathways during steady-state growth, in addition to regulating stable RNA synthesis. This hypothetical layer of regulation would complement control of rRNA transcription, which determines the maximum rate of *steady-state* protein synthesis. For example, studies suggest that basal ppGpp might also regulate the *instantaneous* rate of protein synthesis by limiting purine synthesis (PurF, Wang et al., 2018) or translation cofactor activities (e.g., IF2, Dai et al., 2016) or the fraction of active ribosomes (Zhang et al., 2018). Basal ppGpp might also regulate cell envelope biosynthesis (Noga et al., 2020), as high ppGpp inhibits phospholipid synthesis, and inhibition of phospholipid synthesis arrests peptidoglycan synthesis (Ishiguro et al., 1980; Heath et al., 1994). In this extreme “orchestra conductor” model, ppGpp would not only regulate ribosome abundance, but would also tightly synchronize the synthesis rates of each macromolecule. This model expands the role of ppGpp beyond its better-established role, which is to inhibit general biomass synthesis at starvation-induced concentrations (>600 pmol OD^–1^) like an emergency brake.

Defining the targets of basal ppGpp and identifying how basal ppGpp is maintained are two goals essential to understand how *E. coli* controls growth. To further encourage the recent revival of interest in the mechanisms of steady-state growth regulation and homeostasis (Scott et al., 2010, 2014; Dai et al., 2016), we contribute this Perspective on basal ppGpp in *E. coli* to evaluate the widely assumed notion that ppGpp is always inversely proportional to growth rate.

## Quantifying Basal ppGpp Is Difficult but Essential

Actual ppGpp measurements are essential for determining which cellular processes are watching the growth rate speedometer. The rarity of basal ppGpp measurements is understandable as basal ppGpp is difficult to accurately quantify. The main challenges in measuring basal ppGpp *in vivo* are (1) its low abundance; (2) its chemical instability; (3) the presence of environmentally sensitive enzymes that rapidly hydrolyze and synthesize ppGpp. This means the analytical method must both chemically stabilize ppGpp and immediately denature all enzymes that synthesize or hydrolyze ppGpp. Moreover, in order to study ppGpp dynamics relevant to the rapid ppGpp response (<5 s), the method must enable fast sampling.

Despite these difficulties, several (p)ppGpp measurement methods have been developed, including thin layer chromatography (TLC) (Bochners and Ames, 1982; Sarubbi et al., 1988; Fernández-Coll and Cashel, 2018), high performance liquid chromatography (HPLC) (Ryals et al., 1982; Buckstein et al., 2008; Bokinsky et al., 2013; Varik et al., 2017; Jin et al., 2018) and liquid chromatography mass spectrometry (LC-MS) (Ihara et al., 2015; Patacq et al., 2018). The advantages and disadvantages of each method have summarized in Supplementary Table 1. Whichever method is used, rapid quenching of metabolism during sampling (i.e., no centrifuging of live cells) is required.

## ppGpp Is a Reliable Growth Rate Speedometer: Most Basal ppGpp Measurements Indicate That the Inverse Correlation Between ppGpp and Growth Rate Trends Is Robust

**FIGURE 1:**
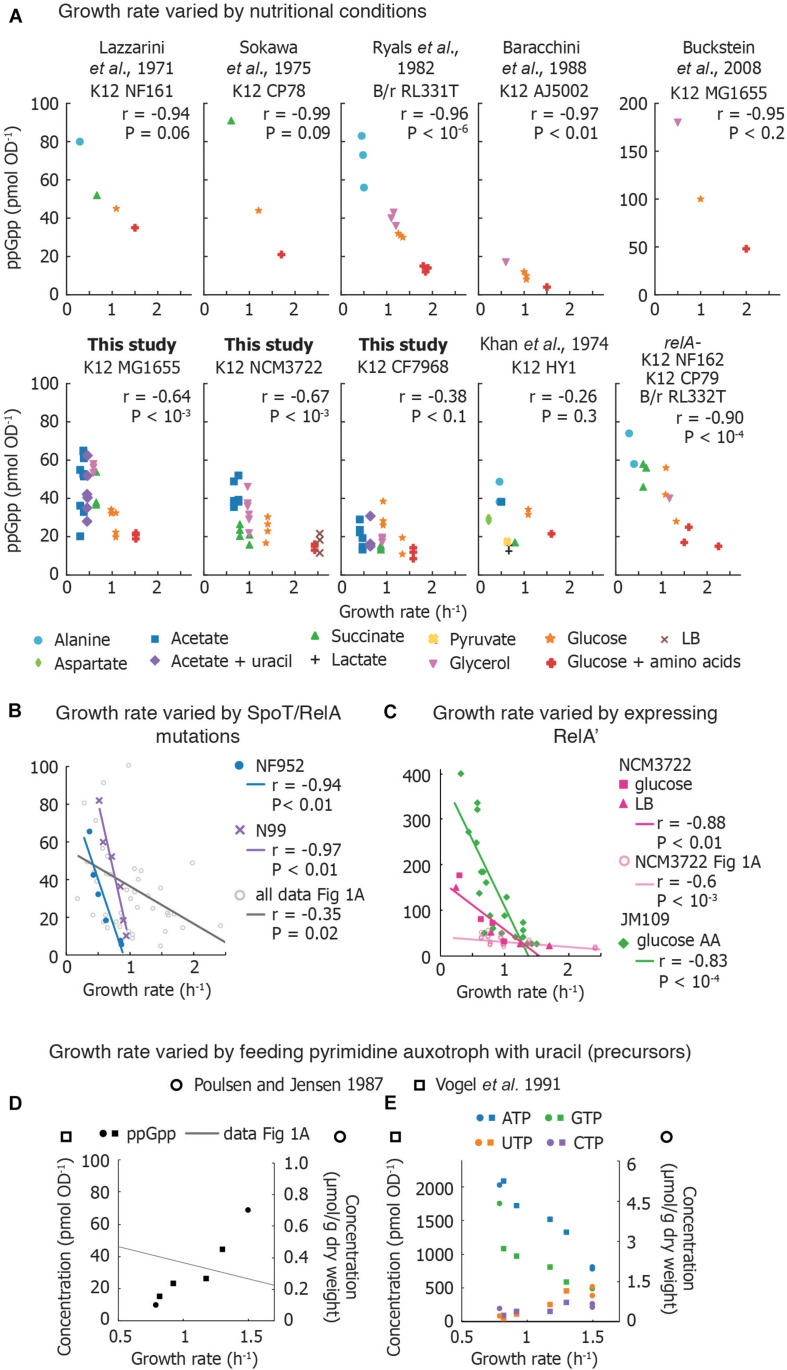
Compilation of measurements of basal ppGpp vs. growth rate. **(A)** Basal ppGpp concentrations measured in *E. coli* strains that generally follow an inverse correlation with growth rate. Both literature data and data obtained for this study are shown (Supplementary Section 3). For data from this study, three technical replicates per biological replicate are shown. For data taken from the literature, the number of biological replicates varies and can be found in Supplementary Material. **(B)** Trends obtained by several *E. coli* strains bearing various mutations in ppGpp synthase/hydrolase enzyme SpoT grown in glucose minimal medium. Each point represents values obtained from one strain. This is compared to compiled data from A. The genotype of each strain used can be found in Supplementary Material. **(C)** Basal ppGpp measured during overexpression of RelA in *E. coli* JM109 (in glucose amino acids without Gln, Glu) and in *E. coli* NCM3722 in LB and glucose minimal medium. **(D,E)** Ribonucleotide concentrations in *E. coli* strains defective in pyrimidine synthesis. **(D)** ppGpp concentrations as a function of growth rate, overlaid with the compiled ppGpp vs. growth rate data of **(A)**, and **(E)** intracellular ATP, GTP, UTP, and CTP concentrations. References to data sets are provided in the Supplementary Material. For **(A–D)**, the Pearson correlation coefficient *r* and significance figure P (from a two-tailed significance test) is shown.

A survey of reported basal ppGpp concentrations combined with our own measurements (Figure 1) indicates that despite apparent variability in absolute concentrations, the inverse correlation with growth rate is robust. We do not include pppGpp measurements, which are less-often reported. All data can be found in the Supplementary Material.

### ppGpp Is an Accurate Growth Rate Speedometer in Wild-Type *E. coli* When Growth Rate Is Varied by Nutrient Source

#### Previously Reported Measurements

Measurements in laboratory-adapted *E. coli* show an inverse correlation between growth rate and basal ppGpp (15–90 pmol OD^–1^) (Figure 1A). Early studies connected basal ppGpp, growth rate, and stable RNA abundance (Lazzarini et al., 1971; Sokawa et al., 1975; Ryals et al., 1982). Khan and Yamazaki (1974) measured basal ppGpp in an *E. coli* patient isolate and found several conditions in which ppGpp concentrations do not align smoothly with the expected trend (Figure 1A). However, RNA/DNA ratios in the outlier cultures followed the expected trend with growth rate (Khan and Yamazaki, 1974).

Reported ppGpp concentrations may differ perhaps due to differences in strains, turbidimeter calibration, or sampling method (Baracchini et al., 1988; Buckstein et al., 2008). Interestingly, even biological replicates show substantial variability (Ryals et al., 1982). Using an assay that exhibited less than 10% variation between individual measurements, Murray and Bremer (1996) report that ppGpp concentrations show 20% variations between biological replicates.

#### New Measurements (This Study)

We measured basal ppGpp concentrations in three *E. coli* K-12 strains using LC-MS (Figure 1A). Concentrations in MG1655 have been reported in Buckstein et al. (2008) for only three conditions (Figure 1A). NCM3722 is becoming increasingly popular as it lacks several genetic defects of MG1655 (Soupene et al., 2003; Brown and Jun, 2015). CF7968 (MG1655 *rph*^+^ but not isogenic with the MG1655 reported here) has been used to demonstrate correlation between the RNA/protein ratio and the growth rate (Potrykus et al., 2011). All three strains exhibit the expected inverse correlation between ppGpp and growth rate. We observed 20% variation in technical replicates from a single culture and 23% average variation between biological replicates, similar to previous reports (Murray and Bremer, 1996).

### RelA Is Not Required to Establish the Correlation Between Basal ppGpp and Growth Rate

At least three studies have compared basal ppGpp concentrations of isogenic *relA*^+^ and *relA*^–^ strains (Lazzarini et al., 1971; Sokawa et al., 1975; Ryals et al., 1982). Each study observed no significant difference in ppGpp concentrations and growth rates in *relA*^+^ and *relA*^–^, indicating that SpoT alone is able to establish an inverse correlation between ppGpp and growth rate (Figure 1A).

#### What Metabolic Process Sets Basal ppGpp Concentrations?

The observation that SpoT is able to maintain the ppGpp-growth rate correlation on its own does not necessarily indicate that RelA activity plays no role in maintaining basal ppGpp. As deacylated tRNA stimulates RelA activity (Haseltine and Block, 1973), one might presume that deacyl-tRNA abundance also correlates inversely with growth rate. However, the aminoacylated fraction of at least six tRNA species remains constant across growth rates. As total tRNA increases in parallel with rRNA, the absolute concentration of deacylated tRNA should also *increase* with growth rate (Dai et al., 2016). There are 43 different tRNA species in *E. coli*, and currently it is not known whether the acylation of each individual tRNA species varies. However, we surmise that RelA may contribute to basal ppGpp as *relA*^–^ mutants exhibit different ribosome pausing behavior than wild-type (Li et al., 2018).

Identifying how SpoT maintains basal ppGpp is essential to understand the metabolic cues that lead to the inverse ppGpp-growth rate correlation. As hydrolysis and synthesis activities of a SpoT homolog are mutually exclusive (Hogg et al., 2004), basal ppGpp is likely set by a balance between the fraction of SpoT proteins engaged in either ppGpp synthesis or ppGpp hydrolysis. Environmental triggers that bias SpoT toward ppGpp synthesis were explored by Murray and Bremer (1996), and included carbon starvation, azide exposure, and simultaneous removal of all 20 amino acids. Although potential regulators have been identified [acyl carrier protein (Battesti and Bouveret, 2006), anti-sigma factor Rsd (Lee et al., 2018) and the small protein YtfK (Germain et al., 2019)], it is unclear how these regulate SpoT.

## Tricking the Growth Rate Speedometer: Artificial Variations of ppGpp Within Fixed Nutritional Conditions

Mutation and overexpression of the *relA* and *spoT* genes enable different ppGpp concentrations in identical nutritional conditions. This also leads to an inverse relationship between ppGpp and growth rate.

### Point Mutations in RelA and SpoT Change Basal ppGpp by Rebalancing Rates of ppGpp Synthesis and Hydrolysis

Non-disabling mutations in *spoT* and *relA* perturb the balance between ppGpp synthesis and hydrolysis, resulting in varied basal ppGpp levels while retaining viability (Sarubbi et al., 1988). Two sets of mutations in different *E. coli* strains (Supplementary Material) exhibited inverse relationships between ppGpp and growth rate that appear to be steeper than the majority of basal level trends observed in various media (Figure 1B). However, as the ppGpp-growth rate relationship was not determined in the parental strains from which these mutants were obtained, direct comparisons with wild-type behaviors are not possible. rRNA decreases as basal ppGpp increases in *spoT* mutant strains as expected (Sarubbi et al., 1988; Hernandez and Bremer, 1990).

### Ectopic Overexpression of RelA Generates a Steep ppGpp-Growth Rate Trendline

Overexpressing the catalytic domain of RelA (referred to as RelA′ or RelA^∗^) artificially elevates ppGpp, inhibits rRNA synthesis and decreases growth rate. Data from ppGpp titrations using RelA′ are compared in Figure 1C. Schreiber et al. (1991) titrated ppGpp concentrations using RelA′ and obtained a ppGpp-growth rate trend that appears steeper than curves obtained for other strains. Two groups recently observed that RelA′ overexpression leads to higher basal ppGpp than expected for a given growth rate. RelA′ overexpression in *E. coli* NCM3722 in both LB medium and glucose minimal medium yields a ppGpp-growth rate curve steeper than the curve obtained in nutrient-limited NCM3722 (confirmed using a Chi-squared test, *P* < 10^–6^) (Zhu et al., 2019; Noga et al., 2020). However, the RNA/protein ratio vs. growth rate trends measured in ppGpp- and carbon-limited cultures closely overlap. This suggests that in the absence of stress, growth rate and RNA synthesis control can be decoupled from *absolute* ppGpp concentrations while still obeying an inverse relationship.

## Breaking the Growth Rate Speedometer: When ppGpp Is Not Inversely Correlated With Growth Rate

### Nucleotide Starvation Causes a Positive Correlation Between Growth Rate and ppGpp

The most dramatic departure from the canonical ppGpp-growth rate trend has been accomplished by disrupting nucleotide metabolism. When the growth rates of nucleotide auxotrophs are titrated by adding limiting nucleotides or nucleotide precursors, ppGpp *increases* in parallel with growth rate. Poulsen and Jensen (1987) first observed this phenomenon in *E. coli* mutants unable to synthesize specific nucleotides [*carAB*- *guaB*(ts)]. When growth rate was titrated with pyrimidine and purine sources, the authors inverted the correlation between ppGpp and growth rate (Figure 1D). Vogel et al. (1991) also found that ppGpp concentrations increased from 15 to 44 pmol OD^–1^ in parallel with growth rate and total RNA in a pyrimidine auxotroph (Figure 1D).

Why does ppGpp correlate positively with growth rate when growth is limited by pyrimidine (uracil) supply? First, uracil limitation does not activate ppGpp synthesis in wild-type strains (Cashel and Gallant, 1969), indicating that RelA and SpoT do not detect all forms of starvation. Second, these auxotrophs exhibit low concentrations of UTP and CTP (Figure 1E) suggesting that ribosome abundance – and thus translation rate – is controlled by substrate concentrations in these mutants (NTP) rather than by inhibitor concentrations (ppGpp). As NTP limitation is relieved, other metabolites likely become limiting for growth, triggering ppGpp synthesis.

### Growth Rate and RNA Content Do Not Strictly Follow ppGpp Concentrations During Out-of-Steady-State Growth Transitions

Steady-state correlations such as the correlation between ppGpp and growth rate imply but do not establish regulatory connections. Hypotheses inspired by correlations must be tested by environmental perturbations. Baracchini and Bremer (1988) monitored growth and rRNA synthesis while adjusting basal ppGpp by adding pseudomonic acid to *E. coli* glucose cultures. Pseudomonic acid causes accumulation of uncharged tRNA and increases ppGpp. High concentrations of pseudomonic acid abruptly increased ppGpp and rapidly arrested growth, consistent with the stringent response. Low concentrations of pseudomonic acid also triggered ppGpp synthesis (up to 60–100 pmol OD^–1^) and an immediate but smaller decrease in rRNA synthesis. However, the instantaneous growth rate (monitored by optical density) was not perturbed in the short term by small increases in ppGpp concentrations.

These out-of-steady-state experiments reveal several important limitations of basal ppGpp regulation. First, the correlation between basal ppGpp and growth rate is broken when growth is out of steady state: were the relationship between growth rate and ppGpp to be as strict during growth transitions as during steady-state growth, a tripling in basal ppGpp would cause an immediate corresponding reduction in growth. Second, unlike the rapid protein synthesis inhibition caused by high ppGpp concentrations (Svitil et al., 1993), small increases of ppGpp (<100 pmol OD^–1^) do not seem to immediately inhibit biomass synthesis (with the exception of stable RNA). This undermines any notion that basal ppGpp directly regulates the instantaneous translation rate. Finally, two additional studies demonstrate that ppGpp and the rate of stable RNA synthesis can be transiently decoupled during nutritional upshifts, suggesting that additional signals may regulate rRNA synthesis (Friesen et al., 1975; Hansen et al., 1975).

## Measurements of Basal ppGpp Reveal That Disruption of ppGpp Binding Sites on RNA Polymerase Does Not Abolish Correlation Between Basal ppGpp, RNA, and Growth Rate

To determine whether RNA polymerase (RNAP) retains regulation by basal ppGpp if its two ppGpp binding sites are disrupted, we measured basal ppGpp levels, growth rates and cellular RNA in *E. coli* strains expressing RNAP mutants (Ross et al., 2013, 2016). Although we did not test a strain bearing both mutations together, we reasoned that mutations in either individual binding site might nevertheless strongly affect RNA synthesis control by basal ppGpp and weaken the relationship between RNA and growth rate, as observed in a ppGpp^0^ strain by Potrykus et al. (2011).

We transferred mutations that disrupt ppGpp binding site 1 [*rpoZ*(wt) *rpoC* R362A R417A K615A; Ross et al., 2013] or that disrupt ppGpp binding site 2 (*rpoC* N680A K681A; Ross et al., 2016) from MG1655 to NCM3722. We confirmed that the stringent response does not arrest RNA synthesis in our mutant strains as strongly as in wild-type (Supplementary Figure 1), qualitatively consistent with results previously observed (Ross et al., 2016). We sampled cultures that had been grown directly from fresh colonies (i.e., without dilution from overnight cultures) to reduce the outgrowth of cells bearing additional RNAP mutations (Murphy and Cashel, 2003).

**FIGURE 2:**
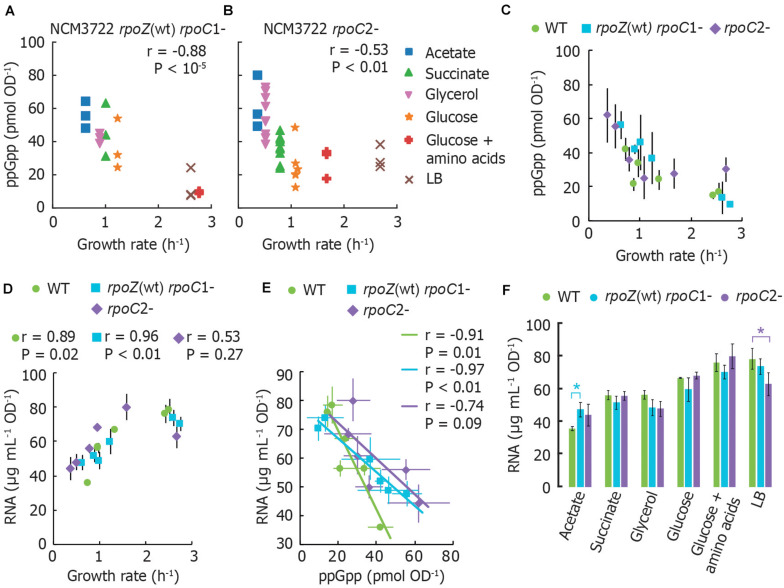
RNA polymerase mutants without ppGpp binding site 1 or 2 still exhibit the typical ppGpp and RNA vs. growth rate trends. **(A)** Basal ppGpp in NCM3722 *rpoZ*(WT) *rpoC*1- and in **(B)** NCM3722 *rpoC*2- (showing technical replicates, 3 per culture). **(C)** Averaged data of A and B overlapped with average NCM3722 wild type data from Figure 1A. **(D–F)** The total RNA concentration of NCM3722 wild type and *rpoC* mutants in various growth media. Error bars represent standard deviations. **(D)** Total RNA concentration plotted as a function of growth rate. **(E)** Data of D plotted as a function of ppGpp concentrations with a linear fit. The average or each condition is shown. See Supplementary Section 3 for materials and methods. **(F)** Data of D plotted to compare RNA content between strains grown in identical media. Bars represent the average of three technical replicates of one culture, with exception of *rpoC*2- in glycerol (2 cultures, 6 replicates) and succinate (3 cultures, 9 replicates). *P < 0.05 for a two-tailed student’s *t*-test. For **(A,B,D,E)**, the Pearson correlation coefficient *r* and significance figure P (from a two-tailed significance test) is shown.

ppGpp concentrations remain inversely correlated with growth rate in both mutants. However, both mutants grow more slowly and have correspondingly higher ppGpp concentrations in most growth media than wild-type NCM3722 (Figures 2A–C). Furthermore, the RNA content of both mutants correlates positively with growth rate, as it does for the wild-type strain (Figure 2D), with exception of the lower RNA concentration for the *rpoC*2- mutant in LB medium. At first glance, this is consistent with the notion that the RNAP mutants are less sensitive to ppGpp, as apparent from the slopes of cellular RNA content vs. ppGpp (Figure 2E). A chi-squared goodness-of-fit test verified that the mutants do not fit the wild-type pattern (*P* < 10^–6^). In other words, higher ppGpp concentrations may be required to inhibit RNA synthesis in these strains. While it might be expected that the cultures expressing ppGpp-insensitive RNAP thus contain a higher RNA abundance than wild-type, we found that for every medium aside from MOPS/acetate, both mutant strains exhibit equivalent or even less RNA per OD unit than does the wild-type (Figure 2F). This is inconsistent with the abolition of growth rate control of RNA content observed in ppGpp^0^ strains (Potrykus et al., 2011).

While our results indicate that neither ppGpp binding site on RNAP is *individually* sufficient to mediate ppGpp control over RNA content, we cannot exclude the possibility that the simultaneous removal of both ppGpp binding sites is required to fully eliminate the ppGpp-RNA content relationship. Other factors may also be implicated in RNA synthesis control in the NCM3722 strain. For instance, TraR, a transcription factor encoded on the F plasmid carried by NCM3722 is known to mimic the action of DksA and ppGpp (Gopalkrishnan et al., 2017).

## Conclusion

We find that the inverse correlation between ppGpp and growth rate during steady-state growth is quite robust, even against removal of the ppGpp synthesis enzyme RelA. Deviations from the trend (e.g., RelA′ overexpression, pyrimidine starvation, and growth transitions) deserve fuller exploration as they likely hint at poorly understood facets of ppGpp biology. Disrupting either individual ppGpp binding site of RNAP did not eliminate the correlation between growth rate, RNA content, and basal ppGpp concentrations. Despite compelling evidence for basal ppGpp control of rRNA synthesis, incorporating ppGpp into quantitative models of cell behavior requires a better understanding of both transcriptional and post-translational targets. In order to advance this goal, we suggest several questions for the field:

1. *What targets are responsive to basal ppGpp concentrations?* As basal ppGpp varies with growth rate in parallel with all biosynthetic fluxes during balanced growth, it is tempting to overextend models of ppGpp control. Targets thought to be regulated during the stringent response may prove insensitive to basal ppGpp. Experiments that monitor ppGpp during growth transitions already suggest that small changes in basal ppGpp do not immediately affect instantaneous protein synthesis or total biomass production. Studies of basal ppGpp concentrations during growth transitions are essential for distinguishing what is influenced by ppGpp. We suggest experiments that monitor protein synthesis during small controlled variations in basal ppGpp (±50 pmol OD^–1^).
2. *What establishes basal ppGpp concentrations?* It is unknown which metabolic cues drive RelA and SpoT to generate basal ppGpp.
3. *What regulates stable RNA synthesis during steady-state growth, aside from basal ppGpp?* Our observation that RNA polymerase mutants lacking either one of the two ppGpp binding sites still exhibit an inverse relationship between RNA content and ppGpp concentrations implies that other factors also regulate RNA content, as has been suggested (Fernández-Coll and Cashel, 2018). However, experiments in a strain simultaneously bearing both RNAP mutations will be necessary to confirm this.
4. *Does basal ppGpp vary inversely with growth rate when growth rate is varied by other nutrients than carbon source?* Measuring basal ppGpp vs. growth rate in conditions that have not yet been tested (e.g., nitrogen source) would also determine whether basal ppGpp is an accurate growth rate speedometer.
5. *Do all species with RSH proteins also maintain basal concentrations of ppGpp (or pppGpp) during steady-state growth?* As RSH proteins are widely distributed (Atkinson et al., 2011), basal ppGpp may be a defining feature of bacterial growth.

We further recommend the use of common reference strains (preferably *E. coli* NCM3722) and defined media to enable comparisons between labs.

## Data Availability Statement

All datasets generated for this study are included in the article/Supplementary Material.

## Author Contributions

NI and GB conceived and designed the experiments and wrote the manuscript. NI performed experiments and data analysis. NI, NB, and MN developed the LC-MS method. All authors contributed to the article and approved the submitted version.

## Conflict of Interest

The authors declare that the research was conducted in the absence of any commercial or financial relationships that could be construed as a potential conflict of interest.
