# Supplementary material for: Calibrating the Bacterial Growth Rate Speedometer: A Re-evaluation of the Relationship Between Basal ppGpp, Growth, and RNA Synthesis in Escherichia coli
Source: Front Microbiol. 2020 Sep 17;11:574872. doi: 10.3389/fmicb.2020.574872 (PMC7527470; doi:10.3389/fmicb.2020.574872)
Supplement: Supplementary file 3 [file Table_3.DOCX]

Supplementary Material

Table S3: Supplementary Figure 1, Supplementary Table 1, and Materials and Methods.

# Supplementary figures

#

**Supplementary Figure 1. Attenuated stringent response in *E. coli* NCM3722 *rpoC*1- and *rpoC*2- mutants**. A) After addition of pseudomonic acid (PA), ppGpp concentrations increase in wild type and both RNA polymerase mutants. B) The total RNA levels stay decrease slightly in the wild type and *rpoC*1- mutant, whereas they mildly increase in the *rpoC*2- mutant. As a control, chloramphenicol (CAM) was added to wild type as chloramphenicol arrests growth without arresting RNA synthesis or elevating ppGpp, a response more similar to the mutant strains.

# A brief overview of current ppGpp analytical methods

The current literature suggests that the effect of ppGpp on cellular processes depends on its intracellular concentration. Slight differences of a few pmol OD^-1^ appear to dramatically affect *E. coli* growth rate. In addition, potential ppGpp levels span a range of less than 10 to over 1000 pmol OD^-1^. In order to understand the role of ppGpp in *E. coli* in both stress conditions as well as steady state growth, it needs to be accurately quantified. Moreover, absolute quantitation is vital as it allows a better comparison of data between research groups as well as quantitative modelling, which helps to understand biological systems.

Here we discuss the methods that have been used to measure (p)ppGpp (Supplementary Table 1). In short, TLC is the oldest method still in use because of its simplicity. It consists of spotting cellular extracts on a membrane, which are separated chromatographically based on charge in one dimension and subsequently based on hydrophobicity in a second perpendicular dimension (Bochners and Ames, 1982). Detection is based on autoradiography and determining the activity in a given spot with scintillation counting. Hence, the culture must be grown in ^32^P phosphate with a constant phosphate uptake rate. This may create a bias in measuring intracellular nucleotide concentrations under conditions with varying ppGpp levels, as ppGpp affects the uptake of phosphate (Irr and Gallant, 1969).

**Supplementary Table 1.** Comparison of current analytical methods for ppGpp quantification.

| Separation + detection method | Equipment needed (excl. sample prep) | Studies with exemplary methods | Basal ppGpp reported? | Sample volume required (for OD ~0.5) | Advantages | Disadvantages |
| --- | --- | --- | --- | --- | --- | --- |
| TLC + autoradio-graphy | TLC development tank, a device to locate UV-absorbing nucleotide spots on the film (e.g. chromato-vue viewing box), X-ray film cassette and intensifying screens, scintillation counter or imaginer | Bochners and Ames, 1982; Sarubbi *et al.*, 1988; Fernández-Coll and Cashel, 2018 | Many studies, for broad range of conditions, (e.g. Lazzarini *et al.,* 1971, Sokawa *et al.*, 1975, Fernández-Coll and Cashel 2018) | 20 µL(Fernández-Coll and Cashel 2018), 200 µL (Sarubbi *et al.*, 1988), 1-2 mL (Lazzarini *et al.,* 1971) | small culture volume, broad scope of nucleotides detectable with single method. | radiolabeling certificate required for both lab and scientist, low phosphate concentration in medium (e.g. 0.2 mM for radiolabeling studies vs. 4 mM in typical growth medium (Bochners and Ames, 1982)) |
| HPLC + UV | HPLC with UV-detector, HPLC column | Ryals *et al.*, 1982; Buckstein *et al.*, 2008; Bokinsky *et al.*, 2013; Varik *et al.*, 2017 | Yes (e.g Ryals *et al.*, 1982; Buckstein *et al.,* 2008; Bokinsky *et al.*, 2013; Varik *et al.*, 2017) | 5-10 mL (Ryals *et al.*, 1982), 50 mL at OD 1 (Buckstein *et al.*, 2008), 10-40 mL (Varik *et al.,* 2017) | HPLC separation does not need to be compatible with MS, so allows non-volatile (high) salt concentrations. | large sample volume. unspecific detection. Baseline separation of compounds is necessary for quantitation. Identification is based on retention time.  High salt concentrations required for elution degrade column performance over time. Difficult to find a single chromatographic method for both NTPs and (p)ppGpp (Varik *et al.,* 2017) |
| HPLC + MS | HPLC, HPLC column, mass spectrometer | Ihara *et al.*, 2015; Patacq *et al.*, 2018 | Twice, but relatively high: 200 pmol OD^-1^ (Ihara *et al.*, 2015) and 0.14 mM (Patacq *et al.*, 2018) | not mentioned (Ihara *et al.*, 2015), 2.8 mL (Patacq *et al.*, 2018) | Higher sensitivity. More accurate detection. Chromatographic separation less vital due to mass-specific detection. Isotope-labelled internal standards reduces errors. Broad scope of nucleotides detectable with single method. | Require MS-compatible buffers. Expensive instrumentation requiring specialized expertise. Sensitive to sample matrix (particularly salts). |

HPLC consists of the separation of nucleotides by applying the sample on a column existing of a solid phase. The column is continuously rinsed with a liquid mobile phase, and depending on the preferred interaction with solid or mobile phase, the different compounds (in this case nucleotides) will travel through the column with different velocities. The separated nucleotides are detected by UV light absorption at the end of the column. Standards of the compounds of interest are necessary to determine the exact time it takes each compound to travel through the column (retention time) and also to make a calibration curve for absolute quantitation of the compound.

LC-MS uses HPLC to separate different compounds, followed by detection via MS instead of UV light. This has several advantages. First, MS is more sensitive (Lu *et al.,* 2017). Secondly, MS allows to detect numerous different compounds at the same time as it rapidly scans for specific masses, whereas with UV light any compound that absorbs at the specified wavelength will give a signal. This means that with HPLC baseline separation of different compounds is vital, as overlapping peaks do not allow quantitation. For the same reason, MS provides more certainty of quantifying a specific compound, while UV detection does not identify exactly the compounds it detects. A disadvantage of MS compared to UV detection is that it is a more expensive, complex and may often prove to be a less robust system that is more prone to technical issues.

Although current methods have successfully characterized basal (p)ppGpp levels in bacteria in multiple conditions, they have several limitations: 1) they require large sample volumes of 10 mL, up to 50 mL for exponentially growing cells) which limits the sample number and time resolution; 2) in most cases they lack absolute quantitation enabling only relative quantitation and 3) they use UV light for detection which is inherently less specific than MS. An ion paring method developed for polar metabolites was able to quantify ppGpp with excellent chromatographic behavior (Coulier et al. 2006). Ihara *et al.* (2015) developed an ion pairing reverse phase ESI QQQ MS/MS method for quantification of ppGpp in plants and *E. coli.* However, the ppGpp concentration in *E. coli* growing in M9 glucose with casamino acids was reported to be about 200 pmol OD^-1^, which is significantly higher than others have measured in this condition. This is almost certainly caused by the cell harvesting method used in the paper, in which live cells were centrifuged before quenching with ice and formic acid. The importance of rapid quenching was also demonstrated by Buckstein *et al.* (2008), who showed that even the shortest pause during the harvesting process will elevate ppGpp and introduce artefacts.

Recently Jin *et al.* (2018) have developed the first UHPLC-HILIC method to separate nucleotides including ppGpp with a limit of detection of 50 nM. This method was tested on algae, which have lower ppGpp levels compared to bacteria (3 pmol g^-1^). The method was however not tested on bacteria, which would be promising. Interestingly, through the use of ion chromatography high resolution MS with double isotopic labeling, Patacq *et al.* (2018) could measure ppGpp as well as pppGpp with correction for degradation of pppGpp into ppGpp during sample preparation. Unfortunately, this method was only used to test the response of *E. coli* to serine hydroxamate, which induces the stringent response and extremely high levels of ppGpp.

Other analytical methods rely on the interaction between Cu^2+^ ions and ppGpp in a colorimetric assay based on modified gold nanoparticles (Chen *et al.,* 2018) or fluorescent silver nanoclusters *(Zhang et al.,* 2013). The ease, low price, lack of complex equipment and speed make these assays very attractive. However, they lack the sensitivity to detect basal ppGpp levels in *B. subtilis* (Chen *et al.,* 2018) or have not been used in a biological matrix(Zhang *et al.,* 2013).

Clearly analytical methods for (p)ppGpp are still being developed and are promising. However, the best test of a ppGpp detection method is not confirmation of high ppGpp during the stringent response, but rather quantification of basal ppGpp in various conditions. Many questions remain unanswered because of these technical limitations. What is the behaviour of ppGpp in different exponential growth or non-stress conditions? What are the dynamics of ppGpp and other nucleotides in changing environmental conditions? Do discrepancies in ppGpp data have a technical or biological (strain-related) origin? As used in this perspective article, the current challenges were overcome with a LC-MS method that allows more sensitive, specific and absolute quantitation than reported so far, with a time resolution of up to 30 s.

# Materials and methods

## Strains and growth conditions

The media used was MOPS-based (8.372 g L^-1^ MOPS, 0.716 g L^-1^ Tricine, 9.5 mM ^14^NH_4_Cl or ^15^NH_4_Cl, 0.276 mM K_2_SO_4_, 0.504 μM CaCl_2_, 0.523 mM MgCl_2_, 50 mM NaCl, 1.2 mM K_2_PO_4_, 10 μM FeSO_4_, 2 μL L^-1^ micronutrient stock (Neidhardt *et al.*, 1974). For growth rate, RNA and LC-MS measurements, all strains were grown as follows: single colonies from fresh LB plates (always streaked from glycerol stocks the previous evening) were grown in a flask aerated using a magnetic stirrer (1200 rpm) within a water bath maintained at 37 °C. This enables rapid sampling without disturbing the culture. Dilution steps from overnight cultures were rigorously avoided to minimize the outgrowth of RNAP mutants (Potrykus et al. 2011). Nevertheless, in follow-up studies the lack of additional mutations should be ascertained by sequencing the strains after experiments are performed.

Strains are listed in Supplementary Table 3. P1 transduction was used to integrate the chromosomal RNA polymerase mutations of *E. coli* RLG14537 (carrying *rpoC*2-) from Ross *et al.*(2016) into the chromosome of *E. coli* NCM3722. Hereto a protocol from the Bob Sauer lab (Moore, 2011) was used and mutations were confirmed by sequencing (Macrogen).

## Total RNA measurement

Cells were sampled and lysed according to the protocol of (Potrykus *et al.,* 2011). Briefly, at an OD of 0.3-0.4, 0.7 OD culture was sampled, kept on ice for a few minutes, and centrifuged at 4000 g and 4 °C for 5 min. Supernatants was removed and cell pellet resuspended in 1 mL ice cold 0.9% NaCl. This was repeated twice. 800 μL cell suspension was added to 200 μL lysis buffer (0.2% SDS in 50 mM EDTA, pH 8). This was incubated at 95 °C for 15 s, followed by rigorous vortexing. Lysates were kept on ice for a few minutes and stored at -20 °C. Subsequent RNA quantitation of the lysates was performed using the Quant-iT^TM^ RNA Assay Kit (Thermo Fisher Scientific) according to manufacturer's instructions.

A chi-squared goodness-of-fit test was used for to compare the linear regressions of RNA vs ppGpp plots of mutants and wild type.

## LC-MS method for ppGpp

### Chemicals

All chemicals for LC-MS purpose (glacial acetic acid, acetonitrile, methanol, formic acid, ammonium acetate) were ULC/MS grade and obtained from Biosolve. Ammonium hydroxide (28-30%) was obtained from Honeywell and acetylacetone (AnalaR Normapur) from VWR. ^15^NH_4_Cl was obtained from CortecNet. UTP, UDP, UMP, CDP and CMP were obtained from VWR. All other chemicals were obtained from Sigma.

### Sampling *E. coli* cultures

An overview of the sampling and sample preparation is presented in figure Supplementary Figure 2 and is based on (Link, Kochanowski, and Sauer 2013). At the time or OD of choice, 1 mL of the culture was pipetted on top of a prewetted filter (0.2 μm pores, 25 mm, Sartorius), placed on a filter manifold under vacuum. The filter is then immediately transferred to a 6-well plate containing 1 mL ice cold 2 M formic acid with the cells facing down to make sure the filter was immediately wetted and cells quenched. Immediately prior to sampling, a known amount of ^14^N internal standards is added to the quenching solution, as this is necessary for absolute quantitation. The composition of the standard mix is shown in Supplementary Table 4.

For cells growing in not isotopically labeled medium (^14^N), a culture grown on ^15^N medium was used as an internal standard. This ^15^N-labeled culture was grown in parallel with the experiment of interest, filtered and resuspended in ice cold 2 M formic acid solution. This 2 M formic acid solution was subsequently used as quenching solution for the unlabeled ^14^N culture. Alternatively, the ^15^N internal standard culture could be neutralized with 27.8 μl 28 % NH_4_OH per mL culture, lyophilized and kept at -80 °C. On the day of use, the ^15^N cell extract would be redissolved in 2 M ice cold formic acid right before use.

**Supplementary Figure 2.** Overview of the analytical method to measure ppGpp and nucleotides in *E. coli*. The bacterial culture was growing on ^15^NH_4_Cl as only nitrogen source. The steps in green are the ones for which variability is corrected for by the presence of ^14^N labeled internal standard (IS). In case nitrogen source was more complex (e.g. amino acids medium), the labeling was reversed, and the internal standard was an extract of another culture grown on ^15^NH_4_Cl.

### Sample preparation

After incubating 30 min to 1 h on ice to extract metabolites from the cells, the filters were washed by holding them with a tweezer and pipetting the quenching solution repeatedly over them. The bottom of the well should also be washed to dislodge any cell debris. The rest of the protocol was based on Ihara *et al.* (2015). The samples were transferred to tubes and neutralized by adding 27.8 μL 28 % NH_4_OH and briefly vortexed. Neutralization is necessary because ppGpp is extralabile in acid (Cashel and Kalbacher, 1970). The samples were stored at -80 °C. The samples were thawed in a water bath at 37 °C for 2 min and subsequently sonicated for 10 min on ice. Then, the samples were centrifuged at 15000 g and 4 °C for 10 min to remove any cellular debris.

Hereafter, solid phase extraction (SPE) was performed to remove sample compounds that might cause so called matrix effects (Dams *et al.,* 2003). Matrix effects include all potential effects of sample molecules that change the chromatographic separation or ionization of a specific analyte of interest (Panuwet *et al.*, 2016). We sought to remove two matrix effects: 1) very strong binding of ppGpp to metal ions, which affects the chromatographic behavior of ppGpp (i.e. affinity for the column); 2) ion suppression: specific matrix compounds suppressing the ionization of the analyte of interest, which decreases sensitivity. Briefly, during electrospray ionization (ESI), the sample is 'sprayed' into tiny charged droplets, in which water and volatile compounds evaporate to leave ionized molecules in the gas phase, which subsequently can be detected by the MS. In various ways, the matrix compounds can deteriorate this process (Panuwet *et al.,* 2016). For example, due to semi-volatile matrix compounds the analyte could precipitate and never reach the gas phase. Matrix compounds can also compete with the analyte to gain charge, preventing its detection. Matrix components that increase the surface tension of the droplets might prevent the droplet break into smaller droplets necessary for reaching the gas phase. Therefore, removal of matrix compounds can greatly improve sensitivity.

Hereto, SPE with a weak anion exchanging capacity was used given that all our compounds of interest (nucleotides) possess acidic phosphate groups (pKa = 0.7-1) (Shabarova and Bogdanov, 2007). The anion exchange occurred at pH 4.5, such that all nucleotides were negatively charged and retained by the column, which is in this range positively charged, whereas all compounds that are neutral or positively charged at this pH (which is the majority of metabolites) would be separated out of the sample (Cohen *et al.,* 2009). The retained nucleotides can be removed from the column by applying a basic solution that renders the solid phase neutral and thereby breaks the electrostatic interaction.

Using SPE presumably also removes metallic cations from the sample. These are of particular interest because these may form a complex with ppGpp, such as Mg^2+^-ppGpp, which are not detected by MS (unless included in the transitions). We verified with MS that these adducts were in fact not present after SPE (data not shown).

Oasis Wax SPE cartridges (Waters, product number 186002489) were equilibrated with first 1 mL methanol and then 1 mL 50 mM ammonium acetate at pH 4.5. After applying the samples, the SPE cartridges were washed with 1 mL 50 mM ammonium acetate pH 4.5, 1 mL methanol, and dried under vacuum for about 5 min. The sample was eluted with 200 μL 5:3:1:1 of methanol:acetonitrile:water:28% NH_4_OH. To this 10 μL 5 % trehalose was added. The trehalose was added under the assumption that it would form hydrogen bonds with ppGpp during the drying (Crowe *et al.*, 1996), which should prevent losses from ppGpp sticking to the tube wall. However, no significant improvement in sensitivity was observed due to trehalose, so this step could be omitted, which was not done for comparability to other data sets acquired within the lab.

After brief vortexing and centrifugating the samples were dried in a vacuum centrifuge for 1 h without heating. Generally no clear or a small transparent pellet was visible. The dried sample was dissolved in 20 μL 5:3:2 methanol:acetonitrile:water. This was centrifuged for 10 min at 15000 g and 4 °C after which 18 μL of supernatant was transferred to a vial.

### LC-MS method

The LC-MS system (Agilent) consisted of a binary pump (G1312B), autosampler (G7167A), temperature-controlled column compartment (G1316A) and triple quadrupole mass spectrometer (G6460C) equipped with a standard ESI source, all operated using MassHunter data acquisition software (version 7.0). 2 μL of the sample was injected onto a iHILIC-fusion column (Hilicon AB, 100 mm length and 2.1 mm internal diameter, 3.5 μm particle size and 100 Å pore diameter) or 3 μL onto a ZIC-cHILIC column (Merck, 100 mm length and 2.1 mm internal diameter, 3 μm particle size and 100 Å pore diameter). For iHILIC the column compartment was set at 20 °C and for cHILIC at 30 °C. Both iHILIC and cHILIC columns were used, but although initial iHILIC columns performed well, batches obtained later from the manufacturer tended to clog easily. Whether this was due to changes to our LC-MS system or due to the manufacturer was not known, but for this reason eventually cHILIC was preferred over iHILIC. Mobile phase A consisted of 3.75 mM ammonium acetate, 1.25 mM acetic acid and 2 mM acetylacetone and mobile phase B of 11.25 mM ammonium acetate, 3.75 mM acetic acid and 2 mM acetylacetone in 80 % acetonitrile. The used gradients and flow rates can be found in Supplementary Table 5. Mass spectrometer operated in dynamic MRM mode (EMV+400) set to unit resolution with 1000 ms cycle time and cell accelerator voltage 4, using transitions defined in Supplementary Table 6. An example chromatogram of separation of standards within a biological sample can be found in Supplementary Figure 3.

LC-MS peak areas were integrated using MassHunter (Agilent). Equation 1 was used to calculate the concentration of compound X in the sample (in pmol OD^-1^), with *^15N^Area_x_* the 15N peak area of compound X, *IS_x_* the amount of compound X in the internal standard in pmol and *OD* the OD of the sample.

$[X]= \frac{{}^{15N}{A{rea}_{x} \times{IS}_{x}}}{{}^{14N}{{Area}_{x} \times OD}}$ (1)

### Remark on units used to quantify ppGpp

Depending on the used analytical method, ppGpp amounts or concentrations have been reported as counts per minute (cpm), peak areas and number of moles, which can be divided by OD, dried cell mass or cell volume, or as ratios of other nucleotides (e.g. ppGpp/ppGpp+GTP). Due to the various units, comparison is sometimes difficult. Volkmer and Heinemann (2011) addressed this by quantifying both the cell size and the number of cells per OD at various growth rates. They observed that the total intracellular volume of all cells for 1 OD unit in 1 mL culture is more or less constant amongst cells in different growth rates and about 3.6 μL. This way mol OD^-1^ data can be calculated from molarity data and vice versa. Varik *et al.* (2017) however did not observe this and calculate molarity from OD data with a different factor.

## Additional tips and tricks for optimal execution of the LC-MS method

What follows are a few more detailed recommendations to optimally execute the method as described above.

1. **The accuracy of internal standards determines the accuracy of the final result.** For each compound, make a dissolved stock from the whole vial of the compound (no weighing using inaccurate or dirty scales), aliquot these and store in the freezer. When making the final internal standard mix, prepare a large volume (some mL) to ideally use a single batch for all experiments. Make the solution to use (close to) maximal accuracy of your pipette and pipette carefully. Aliquot the stock into single-use aliquots. For each experiment, keep what is left of the used internal standard aliquots, such that it can be verified afterwards whether the internal standard was fine.

2. **Keep internal standards on ice.** When using the internal standard aliquots, thaw them right before (e.g. 15 min before usage and leave them on ice). Add the internal standard solution to the 2 M formic acid solution as placing a tiny droplet next to a big drop of 1 mL. Make sure they do not mix yet. Only when the filter containing the cells is placed into the well holding both internal standard and extraction solutions, these will mix. This assures that the internal standard is not degraded by the formic acid solution before the sample touches the formic acid.

3. **Practice the sampling multiple times before sampling**. The key to this protocol is calm yet fast removal of 1 mL culture that is being vigorously stirred, pipetting this on top of a small filter at adequate speed and quickly placing the filter top-down into a 6-well plate holding 1 mL extraction solution. Ideally this step can be done in 5 s. Hereto, make sure

- the pump is set to minimal pressure (or maximal vacuum) and is functioning. Make sure all the tubing connections are air tight. Check with some water if the filtration happens in 1-2 s.
- all tools (filters, pipette, pipette tips, tweezers, waste bag, 6-well plate, OD meter) are arranged such that they can be immediately and easily reached. Put filters in a rack such to be able to instantly grab them with the tweezers. Put already a pipette tip on the pipette. Grab a second 1 mL pipette to measure OD and sample right after one another.
- not to pipette too fast. Due to the aeration of the culture, it is easy to suck up air. Therefore, pay close attention that there are no bubbles in the pipette tip. It is possible to tilt the flask a bit (without affecting the stirring bar) to reach deeper into the culture medium. Also regarding pipetting the 1 mL culture onto the filter, this ideally happens at the same pace as the pulling from the vacuum pump, such that there is no build-up of liquid on top of the filter.
- to not be afraid to tear the filter. The easiest way to remove the filter from the filter manifold (while under vacuum) is to put the tweezers under roughly a 30° angle on top of the filter and then firmly squeeze the ends of the tweezer together. You should be instantly holding the filter.

4. **In each step, try to recover as much sample as possible.** There is an internal standard to correct for losses, but for a nice peak, the LS-MS needs a nice amount of sample. Therefore

- do not leave the sample in the extraction solution for longer than ideally 30 min up to 1 h.
- When the extraction solution (with the precious sample) is removed from the 6-well plate, try to recover as much as possible. Squeeze out the filter with tweezers.
- try to work fast. Do not leave samples unnecessarily waiting on ice. Plan and book all equipment ahead.

5. **Maintenance of the LC-MS is vital.** In our experience, the most challenging part is stability of the column, LC-MS system and ionization efficiency. Therefore, aspire to ideally

- have a liquid chromatography column dedicated to this method only. Clean it after each use, and keep track of the number of runs.
- extensively rinse the whole LC-MS system before usage. Ideally use it right after the source has been thoroughly cleaned. We sometimes observed a clear drop in signal depending on the methods that were used immediately before the run.
- use clean mobile phase flasks each run and prepare buffers fresh. Always use the exact same order for buffer preparations.

**Supplementary Figure 3.** Chromatogram of internal standards (the quantifier ions) from a biological sample run on cHILIC. The amount of internal standard present in the sample is as in Supplementary Table 1, with 25 pmol ppGpp.

**Supplementary Table 3.** Strains used in this study.

| **Strain** | **Description** | **Source** |
| --- | --- | --- |
| *E. coli* DH5α | Used for cloning | Invitrogen |
| *E. coli* NCM3722 | Wild type | CGSC 12355 |
| *E. coli* MG1655 | Wild type | DSMZ 18039 |
| *E. coli* CF7968 | MG1655 *rph*+ | Michael Cashel |
| *E. coli* RLG14536 | MG1655 *rpoZ*Δ2-5-*kan* *rpoC* R362A R417A K615A–*tetAR* (1-2+) | Richard L. Gourse |
| *E. coli* RLG14537 | MG1655 rpoZ(WT)-*kan* *rpoC* N680A K681A –*tetAR* (1+2-) | Richard L. Gourse |
| *E. coli* NCM3722 *rpoC*1- | NCM3722 *rpoC* R362A R417A K615A–*tetAR* (1+2-) | This work |
| *E. coli* NCM3722 *rpoC*2- | NCM3722 *rpoC* N680A K681A-*tetAR* (1+2-) | This work |

**Supplementary Table 4.** Amount of internal standard used for the corresponding compounds added to each *E. coli* sample for quantitation. *For basal and stringent measurements respectively.

| **Compound** | **Amount (pmol)** | **Compound** | **Amount (pmol)** |
| --- | --- | --- | --- |
| ATP | 750 | UTP | 500 |
| ADP | 50 | UDP | 50 |
| AMP | 50 | UMP | 50 |
| GTP | 500 | CTP | 500 |
| GDP | 50 | CDP | 50 |
| GMP | 50 | CMP | 50 |
| ppGpp | 25 or 150* | cAMP | 50 |

**Supplementary Table 5.** Used gradients of the LC-MS methods developed for both iHILIC and cHILIC columns.

| iHILIC | | | cHILIC | | | |
| --- | --- | --- | --- | --- | --- | --- |
| **Time (min)** | **% Mobile phase B** | **Flow rate (mL min^-1^)** | **Time (min)** | **% Mobile phase B** | **Flow rate (mL min^-1^)** |  |
| 0.0 | 100 | 0.3 | 0.0 | 100 | 0.4 |  |
| 0.5 | 100 | 0.3 | 1.0 | 90 | 0.4 |  |
| 1.5 | 85 | 0.3 | 15.0 | 80 | 0.4 |  |
| 10.0 | 85 | 0.3 | 16.0 | 80 | 0.4 |  |
| 10.5 | 85 | 0.25 | 18.0 | 100 | 0.4 |  |
| 13.0 | 30 | 0.25 | 19.0 | 100 | 0.5 |  |
| 15.0 | 30 | 0.25 | 22.0 | 100 | 0.5 |  |
| 17.5 | 100 | 0.25 | 22.5 | 100 | 0.4 |  |
| 20.5 | 100 | 0.25 | 26.5 | 100 | 0.4 |  |
| 21.5 | 100 | 0.3 |  |  |  |  |
| 25.5 | 100 | 0.3 |  |  |  |  |

**Supplementary Table 6.** Transitions and retention times of LC-MS method (cHILIC) for analysis of nucleotides and signalling molecules in *E. coli.* In blue are quantifier ions, in white the qualifier ions.

| **Compound** | **Precursor ion** | **Product ion** | **Retention time (min)** | **Fragmentor** | **Collision energy** |
| --- | --- | --- | --- | --- | --- |
| ADP | 428 | 136.1 | 4.8 | 90 | 40 |
| ADP | 428 | 348.1 | 4.8 | 90 | 10 |
| AMP | 348.1 | 136.1 | 4.3 | 100 | 20 |
| AMP | 348.1 | 119 | 4.3 | 100 | 40 |
| ATP | 508 | 136.1 | 5.3 | 100 | 20 |
| ATP | 508 | 410 | 5.3 | 100 | 10 |
| C2-CoA | 810.1 | 303 | 4.2 | 120 | 29 |
| C2-CoA | 810.1 | 136.1 | 4.2 | 120 | 29 |
| cAMP | 330.1 | 136.3 | 2.3 | 96 | 35 |
| cAMP | 330.1 | 119.1 | 2.3 | 96 | 35 |
| CTP | 484 | 112 | 6.1 | 100 | 10 |
| CTP | 484 | 97 | 6.1 | 100 | 30 |
| GDP | 444 | 152.1 | 5.5 | 96 | 17 |
| GDP | 444 | 135 | 5.5 | 96 | 69 |
| GMP | 364.1 | 152.1 | 5.1 | 96 | 5 |
| GMP | 364.1 | 135 | 5.1 | 96 | 40 |
| GTP | 524 | 152.1 | 6.3 | 122 | 25 |
| GTP | 524 | 135 | 6.3 | 122 | 77 |
| ppGpp | 604 | 152.1 | 6.9 | 142 | 40 |
| ppGpp | 604 | 506 | 6.9 | 142 | 20 |
| UTP | 485 | 97 | 5.7 | 100 | 30 |
| UTP | 485 | 227.1 | 5.7 | 100 | 10 |
| U-15N-ADP | 433 | 141 | 4.8 | 90 | 20 |
| U-15N-ADP | 433 | 353 | 4.8 | 90 | 10 |
| U-15N-AMP | 353.1 | 141 | 4.3 | 100 | 20 |
| U-15N-AMP | 353.1 | 123 | 4.3 | 100 | 40 |
| U-15N-ATP | 513 | 141 | 5.3 | 100 | 20 |
| U-15N-ATP | 513 | 415 | 5.3 | 100 | 10 |
| U-15N-C2-CoA | 817.1 | 305.1 | 4.2 | 120 | 29 |
| U-15N-C2-CoA | 817.1 | 141 | 4.2 | 120 | 29 |
| U-15N-cAMP | 335 | 141 | 2.3 | 96 | 35 |
| U-15N-cAMP | 335 | 124 | 2.3 | 96 | 35 |
| U-15N-CTP | 487 | 115 | 6.1 | 100 | 10 |
| U-15N-CTP | 487 | 97 | 6.1 | 100 | 30 |
| U-15N-GDP | 449 | 157 | 5.5 | 96 | 17 |
| U-15N-GDP | 449 | 139 | 5.5 | 96 | 69 |
| U-15N-GMP | 369.1 | 157 | 5.1 | 96 | 5 |
| U-15N-GMP | 369.1 | 139 | 5.1 | 96 | 40 |
| U-15N-GTP | 529 | 157 | 6.3 | 122 | 25 |
| U-15N-GTP | 529 | 139 | 6.3 | 122 | 77 |
| U-15N-ppGpp | 608.9 | 157 | 6.9 | 142 | 40 |
| U-15N-ppGpp | 608.9 | 511 | 6.9 | 142 | 20 |
| U-15N-UTP | 487 | 97 | 5.7 | 100 | 30 |
| U-15N-UTP | 487 | 229.1 | 5.7 | 100 | 10 |
| U-13C-ADP | 438.1 | 141.1 | 4.8 | 90 | 20 |
| U-13C-ADP | 438.1 | 124.1 | 4.8 | 90 |  |
| U-13C-ATP | 518 | 141.1 | 5.3 | 100 | 20 |
| U-13C-ATP | 518 | 420.1 | 5.3 | 100 | 10 |
| U-13C-GMP | 374.1 | 157.1 | 5.1 | 96 | 5 |
| U-13C-GMP | 374.1 | 140 | 5.1 | 96 | 40 |
| U-13C-GDP | 454.1 | 157.1 | 5.5 | 96 | 17 |
| U-13C-GDP | 454.1 | 140 | 5.5 | 96 | 69 |
| U-13C-GTP | 534 | 157.1 | 6.3 | 122 | 25 |
| U-13C-GTP | 534 | 140 | 6.3 | 122 | 77 |
| U-13C-ppGpp | 614 | 157.1 | 6.9 | 142 | 40 |
| U-13C-ppGpp | 614 | 516 | 6.9 | 142 | 20 |

# References

Bochners, Barry R, and Bruce N Ames. 1982. “Complete Analysis of Cellular Nucleotides by Two-Dimensional Thin Layer Chromato @ Aphy *.” *Journal of Biological Chemistry* 257 (16): 9759–69.

Bokinsky, Gregory, Edward E K Baidoo, Swetha Akella, Helcio Burd, Daniel Weaver, Jorge Alonso-Gutierrez, Héctor García-Martín, Taek Soon Lee, and Jay D Keasling. 2013. “HipA-Triggered Growth Arrest and β-Lactam Tolerance in Escherichia Coli Are Mediated by RelA-Dependent PpGpp Synthesis.” *Journal of Bacteriology* 195 (14): 3173–82. https://doi.org/10.1128/JB.02210-12.

Buckstein, Michael H, Jian He, and Harvey Rubin. 2008. “Characterization of Nucleotide Pools as a Function of Physiological State in \textit{Escherichia Coli}.” *Journal of Bacteriology* 190 (2): 718–26.

Cashel, Michael, and Barbara Kalbacher. 1970. “The Control of Ribonucleic Acid Synthesis in \textit{Escherichica Coli}. V. Characterization of a Nucleotide Associated with the Stringent Response.” *The Journal of Biological Chemistry* 245 (9): 2309–18.

Chen, Jia, Yanni Huang, Xiaoyan Yang, Haijuan Zhang, Zhan Li, Bo Qin, Xingguo Chen, and Hongdeng Qiu. 2018. “Highly Sensitive and Visual Detection of Guanosine 3′-Diphosphate-5′-Di(Tri)Phosphate (PpGpp) in Bacteria Based on Copper Ions-Mediated 4-Mercaptobenzoic Acid Modified Gold Nanoparticles.” *Analytica Chimica Acta* 1023: 89–95. https://doi.org/10.1016/j.aca.2018.02.082.

Cohen, S, M Megherbi, L P Jordheim, I Lefebvre, C Perigaud, C Dumontet, and J Guitton. 2009. “Simultaneous Analysis of Eight Nucleoside Triphosphates in Cell Lines by Liquid Chromatography Coupled with Tandem Mass Spectrometry.” *Journal of Chromatography B* 877 (30): 3831–40.

Coulier, Leon, Richard Bas, Sonja Jespersen, Elwin Verheij, Mariët J. Van Der Werf, and Thomas Hankemeier. 2006. “Simultaneous Quantitative Analysis of Metabolites Using Ion-Pair Liquid Chromatography-Electrospray Ionization Mass Spectrometry.” *Analytical Chemistry* 78 (18): 6573–82. https://doi.org/10.1021/ac0607616.

Crowe, L M, D S Reid, and J H Crowe. 1996. “Is Trehalose Special for Preserving Dry Biomaterials?” *Biophysical Journal* 71: 2087–93.

Dams, Riet, Marilyn A Huestis, Willy E Lambert, and Constance M Murphy. 2003. “Matrix Effect in Bio-Analysis of Illicit Drugs with {LC}-{MS}/{MS}: Influence of Ionization Type, Sample Preparation, and Biofluid.” *Journal of the American Society for Mass Spectrometry* 14 (11): 1290–94.

Fernández-Coll, Llorenç, and Michael Cashel. 2018. “Contributions of SpoT Hydrolase, SpoT Synthetase, and RelA Synthetase to Carbon Source Diauxic Growth Transitions in \textit{Escherichia Coli}.” *Frontiers in Microbiology* 9 (AUG): 1–13.

Ihara, Yuta, Hiroyuki Ohta, and Shinji Masuda. 2015. “A Highly Sensitive Quantification Method for the Accumulation of Alarmone PpGpp in Arabidopsis Thaliana Using UPLC-ESI-QMS/MS.” *Journal of Plant Research* 128 (3): 511–18. https://doi.org/10.1007/s10265-015-0711-1.

Irr, Joseph, and Jonathan Gallant. 1969. “The Control of Ribonucleic Escherichia Coli Synthesis In.” *Journal of Biological Chemistry* 244 (8): 2233–39.

Jin, Hui, Yong Min Lao, Jin Zhou, Huai Jin Zhang, and Zhong Hua Cai. 2018. “A Rapid UHPLC-HILIC Method for Algal Guanosine 5′-Diphosphate 3′-Diphosphate (PpGpp) and the Potential Separation Mechanism.” *Journal of Chromatography B: Analytical Technologies in the Biomedical and Life Sciences* 1096 (February): 143–53. https://doi.org/10.1016/j.jchromb.2018.08.009.

Link, H, K Kochanowski, and U Sauer. 2013. “Systematic Identification of Allosteric Protein-Metabolite Interactions That Control Enzyme Activity in Vivo.” *Nature Biotechnology* 31 (4): 357–61. https://doi.org/10.1038/nbt.2489.

Lu, Wenyun, Xiaoyang Su, Matthias S. Klein, Ian A. Lewis, Oliver Fiehn, and Joshua D. Rabinowitz. 2017. “Metabolite Measurement: Pitfalls to Avoid and Practices to Follow.” *Annual Review of Biochemistry* 86 (1): 277–304. https://doi.org/10.1146/annurev-biochem-061516-044952.

Moore, Sean. 2011. “Sauer: P1vir Phage Transduction.” Edited by OpenWetWare.

Neidhardt, F C, P L Bloch, and D F Smith. 1974. “Culture Medium for Enterobacteria.” *Journal of Bacteriology* 119 (3): 736–47.

Panuwet, Parinya, Ronald E Hunter Jr, Priya E D Souza, Xianyu Chen, A Radford, Jordan R Cohen, M Elizabeth Marder, Kostya Kartavenka, P Barry, and Dana Boyd Barr. 2016. “Biological Matrix Effects in Quantitative Tandem Mass Spectrometry-Based Analytical Methods: Advanced Biomonitoring.” *Critical Review in Analytical Chemistry* 46 (2): 93–105. https://doi.org/10.1080/10408347.2014.980775.Biological.

Patacq, Clément, Nicolas Chaudet, and Fabien Létisse. 2018. “Absolute Quantification of PpGpp and PppGpp by Double-Spike Isotope Dilution Ion Chromatography-High-Resolution Mass Spectrometry.” *Analytical Chemistry* 90 (18): 10715–23. https://doi.org/10.1021/acs.analchem.8b00829.

Potrykus, K, H Murphy, N Philippe, and M Cashel. 2011. “PpGpp Is the Major Source of Growth Rate Control in E-Coli.” *Environmental Microbiology* 13 (3): 563–75. https://doi.org/DOI 10.1111/j.1462-2920.2010.02357.x.

Ross, Wilma, Patricia Sanchez-Vazquez, Albert Y. Chen, Jeong Hyun Lee, Hector L. Burgos, and Richard L. Gourse. 2016. “PpGpp Binding to a Site at the RNAP-DksA Interface Accounts for Its Dramatic Effects on Transcription Initiation during the Stringent Response.” *Molecular Cell* 62 (6): 811–23. https://doi.org/10.1016/j.molcel.2016.04.029.

Ryals, J, R Little, and H Jbremer. 1982. “Control of RNA Synthesis in Escherichia Coli After a Shift to Higher Temperature” 151 (3): 1425–32.

Sarubbi, Edoardo, Kenneth E. Rudd, and Michael Cashel. 1988. “Basal PpGpp Level Adjustment Shown by New SpoT Mutants Affect Steady State Growth Rates and RrnA Ribosomal Promoter Regulation in Escherichia Coli.” *MGG Molecular & General Genetics* 213 (2–3): 214–22. https://doi.org/10.1007/BF00339584.

Shabarova, Z, and A Bogdanov. 2007. “Structure of Nucleotides.” In *Advanced Organic Chemistry of Nucleic Acids*, 71–92. Wiley-{VCH} Verlag {GmbH}.

Varik, Vallo, Sofia Raquel Alves Oliveira, Vasili Hauryliuk, Tanel Tenson, Sofia Raquel, Alves Oliveira, and Vasili Hauryliuk. 2017. “HPLC-Based Quantification of Bacterial Housekeeping Nucleotides and Alarmone Messengers PpGpp and PppGpp.” *Scientific Reports* 7 (1): 1–46. https://doi.org/10.1038/s41598-017-10988-6.

Volkmer, Benjamin, and Matthias Heinemann. 2011. “Condition-Dependent Cell Volume and Concentration of Escherichia Coli to Facilitate Data Conversion for Systems Biology Modeling.” *PLoS ONE* 6 (7): 1–6. https://doi.org/10.1371/journal.pone.0023126.

Zhang, Pu, Yi Wang, Yong Chang, Zu Hong Xiong, and Cheng Zhi Huang. 2013. “Highly Selective Detection of Bacterial Alarmone PpGpp with an Off-on Fluorescent Probe of Copper-Mediated Silver Nanoclusters.” *Biosensors and Bioelectronics* 49: 433–37. https://doi.org/10.1016/j.bios.2013.05.056.
